# Is Leprosy Infectious?

**Published:** 1889-06-22

**Authors:** 


### Is Leprosy Infectious?

Several recent occurrences have tended to bring this question into prominence. Some time ago, as noticed in the *Hospital*, December 8 last, the Rev. H. P. Wright, rector of Greatham, " made known to the world at large the important and highly interesting fact that at last it had been shown by a terrible experiment on a criminal condemned to death that leprosy may be conveyed by inoculation," a fact which, in the Rev. Mr. Wright's opinion, goes far to prove that the malady is contagious. Still more recently we were informed of the death of Father Damien, who heroically devoted himself to the service of the lepers in the leper settlement of the Hawaiian Archipelago, after a time contracting the disease and dying. Just now there is a paragraph " going the round of the press," and representing that an old soldier in Ireland being a leper, a proposal was made to transfer him to some leper asylum in India. But the military authorities would have nothing to do with him, and no vessel could be found wherein he would be received as a passenger.

Before considering the question of infection it is necessary to have a clear definition of the term, for the general public are apt to confuse infection with contagion, whereas the two terms have distinct and separate meanings. By infection should be understood communication of disease through a pestiferous or contaminated atmosphere. By contagion should be understood communication of disease by a specific virus originating within the body. The simplest kind of contagion is that where a disease is communicated in one way only—viz., by the transmission of poisonous matter conveyed from the body of the sick, and applied to a healthy person, either immediately or through the medium of anything which has been in contact with the sick. There are certain maladies which are only contagious —that is, communicated by direct agency. There are other diseases which are communicated through the medium of the atmosphere or by indirect agency. And there are certain maladies which are both infectious and contagious. The question is, to which of these three classes does leprosy belong?

Now, although the Rev. Mr. Wright stated that by a terrible experiment on a criminal it had *at last* been proved that leprosy was contagious, there was no necessity for any such proof, for the fact had long been known. Damsch long since proved by experiment that leprosy may be communicated to animals by inoculation. There is also on record the well authenticated case of a boy pricking himself with a needle which a leper had used, from which injury leprosy developed. The fact that leprosy commences most frequently on exposed parts, especially the hands and feet, encourages the conclusion of contagion from the outside. As with many other diseases so with leprosy, a microbe has been discovered in the leprous tissue. Whether this is a cause or a consequence of the malady cannot at present be confidently asserted. But whether or not the poison of leprosy be an organised microbe has little or nothing to do with the question of contagion. All that is required is the transmission of leprous matter to the healthy body, and an infinitesimal portion of leprous poison is sufficient. But in order that the poison may act, it seems necessary that it should come into contact with the abraded skin. Although leprosy prevails in some European countries, it may be doubted if it is the same inveterate form of the malady met with in the East, and it is certainly not common in any European country. For this there are several reasons, the one applying to the present argument being that Easterns do not usually wear shoes and stockings, the poorer classes, among whom leprosy prevails most, never using such articles of apparel. A person of the class referred to without some more or less slight abrasion on or about the foot is almost an exception. If he happens to be in the neighbourhood of a leper with open sores, the accidental transference of a particle of leprous discharge to the sore or abrasion on the person of the healthy individual, may occur in very many different ways. We have not space to parade all the arguments which might be adduced as to the spread of leprosy as above sketched, but we are convinced that the weight of evidence is in favour of such dissemination of the malady.

As regards infection or communication of the disease through the medium of the atmosphere, we hold that evidence is against such transmission. There are innumerable instances of persons living near, or even with, lepers for years, and not contracting the disease. In all human probability Father Damien contracted his leprosy accidentally, from some abrasion of his skin coming into accidental contact with leprous discharge. If leprosy were infectious, that is, spreading through the medium of the atmosphere in the manner in which influenza, measles, or scarlet fever may be disseminatad—leprosy would be a still more prevalent malady than it is, even in the East. There leprosy is pretty much confined to leper's families, or to districts, which tends to confirm the view that leprosy is spread first by hereditary transmission, and, secondly, by accidental contact of leprous poison with an abraded skin.

There is scarcely any matter, however, on which more difference of opinion has been expressed than on the mode in which leprosy is spread. We only profess to give as a conclusion that to which we are led by, as we think, the weight of evidence, combined, however, with some personal experience in connection with the malady. Those who have seen most of leprosy are opposed to the view of the disease being infectious, distinctly stating that it is not communicable through the atmosphere. Thus, Dr. Robert Liveing states there is at present no evidence to show that leprosy is contagious in the ordinary sense of the word, which ordinary sense in public acceptation means also infection. Dr. Rastonyee Khory, of Bombay, in his work on practice of medicine, states it is not infectious. Dr. Vandyke Carter is of opinion that it is only contagious by the direct or indirect conveyance of the disease from person to person. Sir William Moore states : " The propagation of leprosy must take place by contagion through the abraded skin. This explains the reason why so many escape although in constant communication with lepers, whereas accidental contact with leprosy poison with wounded skin explains many cases which do occur." It is true that the reports of Danielson, Boeck, Virchow, and of the English College of Physicians, which are so frequently quoted in connection with the subject, tend to prove the heredity and not the contagiousness of leprosy. But facts regarding contagion have since been recorded which, in our opinion, without negativing the conclusions of heredity being a cause, supply also another factor on direct communication.

Leprosy was at one period very common in Great Britain, although it is doubtful if it ever assumed the destructive form of Indian leprosy. The knowledge of what has been renders some persons as the Rev. Mr. Wright fearful of what may be. We are not prepared to say that leprosy cannot spread in England, but we certainly think thatit i most unlikely to do so from the few centres which exis. In Eastern countries very few of the European residents contract leprosy, the reasons given being that they do not come of leper families, that they wear shoes and stockings, that their habits are cleanly, and that they are therefore less liable to become accidentally inoculated. It must be recollected that leprosy, like various other diseases, flourishes best under bad sanitary and bad personal hygienic conditions. When leprosy prevailed in England, sanitation and hygiene were unknown. People had rotting straw on their floors instead of carpets, and every habitation had its adjacent cesspool. We may not yet have attained Dr. Richardson's standard of perfection. But enough has been done to render the spread of leprosy most improbable, if not impossible. We do not discern the least reason for any scare or anxiety on the subject. Doubtless a leper with open sores should be isolated, or at least means should be taken to prevent the accidental transference of discharge from such sores to healthy people. But before the leprosy progresses into sores, we hold that a leper is harmless to the public, and cannot disseminate the disease. We are also of opinion that lepers should not marry. This they are not likely to do in England, but in the East they are not so particular. This is a question, however, which concerns Eastern Governments. In conclusion, we may mention, with reference to the Irish soldier leper referred to above, we are of opinion that if there were no open sores he might have been safely conveyed in any ship, and that if there were open sores he might have been safely conveyed under suitable precautions. The proposal, however, to convey him to an Indian leper asylum is scarcely feasible. In the first place, a tropical climate would certainly aggravate and increase the rapidity of the progress of his leprosy, and, secondly, we are not aware of any Indian leper asylum in which Europeans are received.

